# Improved evidence to guide dementia care is urgently needed

**DOI:** 10.1073/pnas.2312272120

**Published:** 2023-08-22

**Authors:** Andrew N. March, Clare Stroud, Eric B. Larson

**Affiliations:** ^a^Health and Medicine Division, National Academies of Sciences, Engineering, and Medicine, Washington, DC 20001; ^b^Department of Medicine, University of Washington, Seattle, WA 98195

The approvals of aducanumab (1) in 2021 and lecanemab (2) in 2023 for the treatment of Alzheimer’s disease have garnered a great deal of attention within the dementia community. Press releases highlighting other drugs in development aiming to reduce amyloid plaques—which are thought to contribute to dementia symptoms—such as donanemab (3) continue to raise hope for more curative treatments. The arrival of these new drugs continues the decades-long process in search of treatments and cures for Alzheimer’s disease and related dementias. However, available treatments have the potential to cause serious side effects, and it remains unclear whether a causal relationship exists between dementia symptoms and the amyloid plaques that the drugs target. Citing the risk of side effects and the relatively small clinical improvement in the symptoms, many researchers and clinicians have urged caution in prescribing these new treatments (4, 5). Moreover, given that innovator drugs take an average of 9 y to develop (6), reliance on finding a pharmaceutical solution risks ignoring the needs of the millions of people currently living with dementia. While more effective treatments are developed and someday may become more widely accessible, interventions that promote the personhood and well-being of persons living with dementia—as well as that of their care partners and caregivers—will remain integral to dementia care.

Millions of individuals across the United States and around the world are living with dementia (7). Persons living with dementia can lead rewarding and fulfilling lives, and to do so, they need medical care, physical quality of life, social and emotional quality of life, and access to services and supports. To meet these needs, persons living with dementia may comanage with or rely on care partners or caregivers for adequate care and support. Many care partners and caregivers report positive benefits from assuming that role (8). However, the potential for negative consequences for their health, relationships, and finances necessitates a system of supports and services for the care partner and caregiver as well.

Fortunately, the standard of care for persons living with dementia has advanced past harmful practices of using physical restraints and high doses of antipsychotic medications to exert control over patients. Hundreds of interventions for persons living with dementia, their care partners, and caregivers have been developed and tested in randomized clinical trials. To evaluate which of these interventions should be considered for broad implementation, at the request of the National Institute on Aging (NIA), the National Academies of Sciences, Engineering, and Medicine convened the Committee on Care Interventions for Individuals with Dementia and Their Caregivers in 2018. The committee included experts in dementia care and aging research, health policy, and research methods. They met several times between 2018 and 2020 to gather evidence and deliberate as a group, in addition to holding a virtual public workshop in April 2020. In 2021, the committee released its final consensus report, Meeting the Challenge for Persons Living with Dementia and Their Care Partners and Caregivers: A Way Forward (9).

## A Framework for Providing Care

Dementia can result from a variety of neurodegenerative diseases and may present differently in different individuals and at different stages of disease progression. Similarly, the multitude of care partner and caregiver arrangements typically change over time. Despite the dynamic nature of dementia, the committee identified six guiding principles for dementia care, services, and supports that are universally applicable (Box 1). The guiding principles emphasize the importance of recognizing persons living with dementia, their care partners, and caregivers as individuals with unique needs that deserve to be met regardless of individual or community circumstances. Institutions and organizations that provide dementia care should use these guiding principles as they provide care and as driving forces for continuous quality improvement efforts to improve the content and reach of their services.

Box 1. Guiding principles for dementia care, services, and supports

1. Person-centeredness

2. Promotion of well-being

3. Respect and dignity

4. Justice

5. Racial, ethnic, sexual, cultural, and linguistic inclusivity

6. Accessibility and affordability

Recognizing the insufficient access to quality dementia care (10), especially for individuals from racial and ethnic minority groups (11), the committee also outlined a set of core components of care, services, and support (Box 2). Implementation of interventions that incorporate these core components would represent a marked improvement in the quality of care provided to persons living with dementia, their care partners, and caregivers. The committee emphasized that individuals should have the opportunity to inform how the components of care will be implemented within personalized care plans, which typically need to be adapted as the disease progresses with ongoing input from the individuals involved.

Box 2. Core components of care, services, and supports for persons living with dementia, care partners, and caregivers

1. Detection and diagnosis

2. Assessment of symptoms to inform planning and deliver care

3. Information and education

4. Medical management

5. Support in activities of daily living

6. Support for care partners and caregivers

7. Communication and collaboration

8. Coordination of medical care, long-term services and supports, and community-based services and supports

9. Supportive and safe environment

10. Advance care planning and end-of-life care

**Fig. 1.**
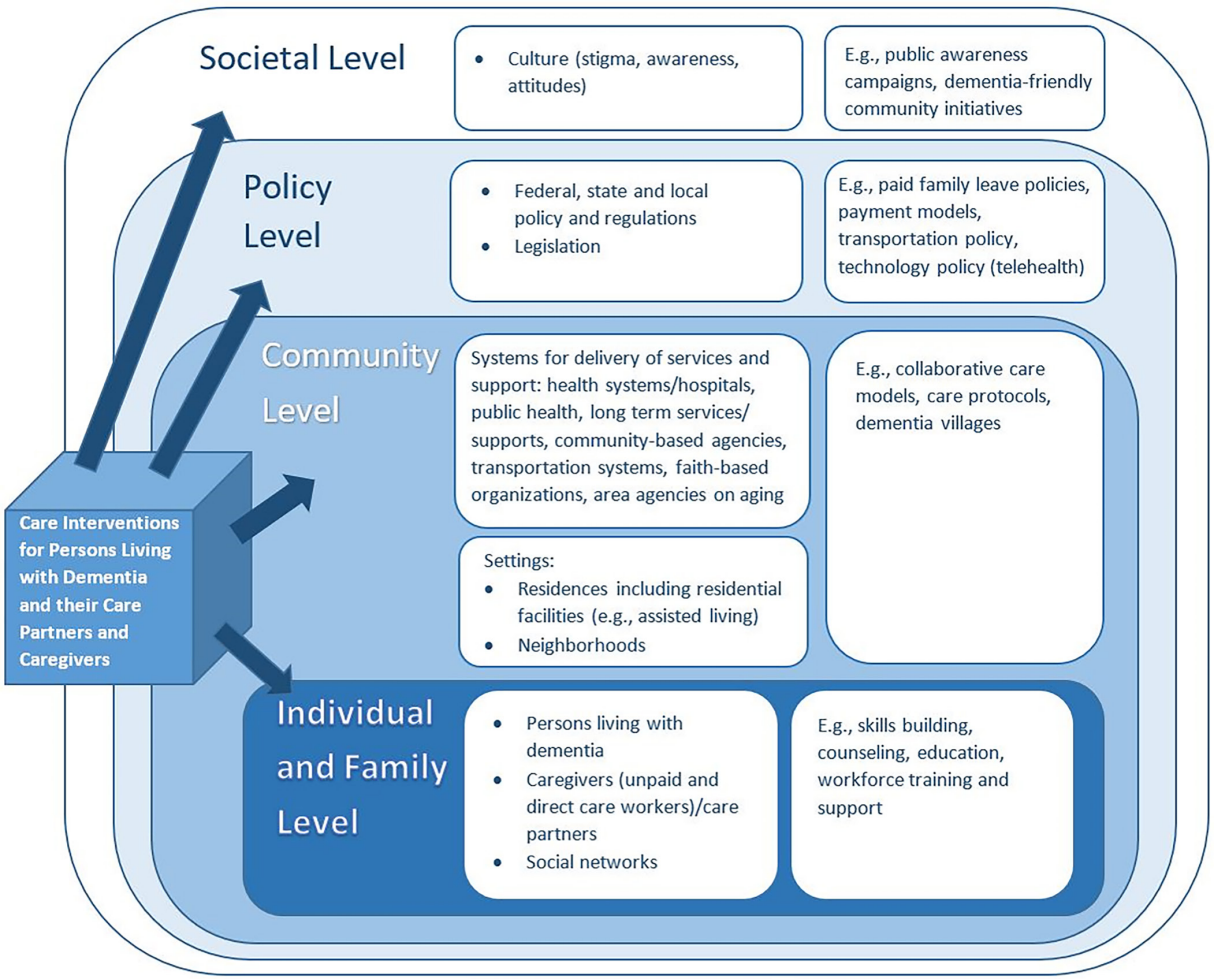
Framework of dementia care interventions. Reprinted with permission from ref. 9.

While many dementia care interventions are targeted to the individual living with dementia, their care partner, or caregiver, there are various levels into which most dementia care interventions can be categorized (12). A framework for care interventions was adapted from a 2016 National Academies report, Families Caring for an Aging America (8), to systematically categorize dementia care interventions by the settings, networks, and environments in which they are implemented (Fig. 1). The intervention levels are nested within one another, representing that higher levels naturally encompass lower levels within the framework. The framework should serve as a tool for researchers and decision makers who set research priorities, enabling evaluation of interventions within and across levels, as well as identifying levels that receive insufficient focus and resources.

## Promising Care Interventions

The evidence base for care interventions for persons living with dementia and their care partners and caregivers has evolved over decades and, like efforts to find pharmaceutical solutions, is vast. However, the ability to confidently draw conclusions from it is limited by small sample sizes, a lack of common outcome measurements, short-duration studies, and a lack of participant diversity (12). Despite these challenges, two interventions have sufficient evidence to merit further study and dissemination and implementation with evaluation. Collaborative care models and multicomponent interventions for informal caregivers should be implemented in real-world settings with continued monitoring and evaluation, quality improvement, and information sharing.

A systematic review from the Agency for Healthcare Research and Quality (AHRQ) (12), for which the committee provided input on the design, informed the committee on the strength of evidence for dementia care interventions available in the published scientific literature. Two interventions—collaborative care models and REACH (Resources for Enhancing Alzheimer Caregiver Health) II—demonstrated consistent evidence of benefit and showed a favorable profile for equity, acceptability, feasibility, and resource considerations when analyzed through the GRADE Evidence to Decision Framework (13).

Collaborative care models can be defined as interventions that leverage multidisciplinary teams and a combination of medical and psychosocial approaches in the care of persons living with dementia. Collaborative care models, including ACCESS and Care Ecosystem models, demonstrated improvement in quality of life for persons living with dementia and system-level markers such as the number of emergency department visits (12). The AHRQ review also found inconsistent evidence that collaborative care models may improve neuropsychiatric symptoms and reduce nursing home placement for persons living with dementia, reduce strain, and depression in care partners and caregivers, as well as improve their adherence to dementia care guidelines.

Multicomponent interventions are a broad category that encompasses interventions composed of multiple components. REACH II is a particular example of a multicomponent intervention and was the only specific multicomponent intervention model—along with its adaptations—identified in the AHRQ report that had a reliable trend toward benefit. REACH II comprises seven core components:

1. Problem-solving
2. Skills training
3. Stress management
4. Support groups
5. Provision of information
6. Didactic instruction
7. Roleplaying

Multicomponent interventions for informal caregivers, specifically REACH II and its adaptations, improved caregiver/care partner depression at 6-mo follow-up (12). Several of the individual REACH II and REACH II adaptation studies suggest a reduction in caregiver strain or stress, challenging behaviors in persons living with dementia, caregiver frustration or bother, and physical symptoms of psychiatric conditions, in addition to improvements in self-reported social support, self-reported caregiver health, caregiver reactions to challenging behaviors, positive aspects of caregiving, and safety of persons living with dementia.

## The Road Ahead

To move the evidence base forward with actionable results, the methodology of future studies should be robust enough to create confidence regarding effectiveness within studies and comparisons between studies. Consideration should be given to whether studies are sufficiently powered to make statistically significant comparisons between groups. The field should develop a common set of outcomes to be measured—prioritizing outcomes most important to persons living with dementia, care partners, and caregivers—to facilitate comparisons between studies. To capture the effects of interventions across the course of disease progression, research should also include longer-term studies and follow-up of participants.

Future studies should recruit participants that reflect the diversity of persons living with dementia, their care partners, and caregivers. This includes individuals from racial and ethnic minority groups LGBTQIA+ individuals, people with disabilities, and rural communities. Research that fails to capture the diversity of the target population not only generates biased results but furthers health inequities (14). From the very beginning of study conception, sponsors and researchers should develop a plan for recruitment and retention of underrepresented groups, which is now required through Diversity Action Plans (15). Studies will benefit from relationships with diverse community organizations that are developed and nurtured long before recruitment begins.

The NIA IMbedded Pragmatic Alzheimer’s Disease (AD) and AD-Related Dementias Clinical Trials (IMPACT) Collaboratory is designed to support and advance pragmatic, translational research in dementia care (16). This model of embedded pragmatic clinical trials aims to speed the translation of research into practice. The IMPACT Collaboratory has contributed insights into the real-world implementation of dementia care interventions through original research (17, 18), research infrastructure, training resources, development of a stronger investigator cadre, and innovative methods research.

Advancing care for persons living with dementia, their care partners, and caregivers—alongside the efforts underway to find pharmaceutical treatments that alter the course of disease—are endeavors worthy of ongoing investment. Modern cancer care demonstrates how pharmaceutical and care interventions together benefit people living with the disease and are likely to be invaluable to dementia care as well. Radiation therapy and chemotherapy changed the course of cancer care, ultimately benefiting millions of persons and saving lives. Over time, these medical advances were paired with improvements to the care that those living with cancer receive during the course of treatment. Research advances in care delivery paved the way to improved cancer care. The dementia field similarly needs convincing evidence to guide care improvements and better provide for the needs of people living with dementia. Despite continued hope for a cure for Alzheimer’s disease and related dementias—even the most attractive pharmaceutical solutions do not yet hold the promise of curing diseases. There is a more urgent need for improved care in how persons living with dementia and their care partners and caregivers experience dementia. Millions will benefit from improved care now.

Sustained resources and dedication are needed to continue testing dementia care interventions and moving successful interventions into real-world care settings for implementation and continued evaluation. Persons living with dementia, their care partners, and caregivers have long demanded and desperately deserve the basic quality of care and well-being that this research can enable. With recent advances in research methods and infrastructure, a future in which people can live well with dementia is coming into sharper focus.
